# Fast simultaneous estimation of *n*D transport coefficients and source function in perturbation experiments

**DOI:** 10.1038/s41598-023-30337-0

**Published:** 2023-02-24

**Authors:** Ricky van Kampen, Jelle de Vries, Siep Weiland, Marco de Baar, Matthijs van Berkel

**Affiliations:** 1 DIFFER - Dutch Institute for Fundamental Energy Research, 5612 AJ Eindhoven, The Netherlands; 2 Department of Mechanical Engineering, Eindhoven University of Technology, 5600 MB Eindhoven, The Netherlands; 3 Department of Applied Physics, Eindhoven University of Technology, 5600 MB Eindhoven, The Netherlands; 4 Department of Electrical Engineering, Eindhoven University of Technology, 5600 MB Eindhoven, The Netherlands

**Keywords:** Mechanical engineering, Magnetically confined plasmas, Hydrology

## Abstract

In the calculation of transport coefficients from experimental data precise knowledge of the source is usually assumed, while the identification of the coefficients focuses on specific geometries and one spatial variable. This paper presents a method for the simultaneous estimation of both the distributions of transport coefficients as well as the source profile. A convex solution of the inverse problem is retained which makes the calculations highly computational efficient. Moreover, this allows for the estimation of multi-dimensional transport coefficients, source terms, and in the future the analysis of the effect of regularization on experimental data and transport coefficient distributions.

## Introduction

In many physical systems, generic scalar transport determines the behavior. Examples are transport of particles, heat and momentum in nuclear fusion reactors^1^, the hyporheic flow in groundwater–surface interaction systems^2^, thermal transport for medical treatments such as local hyperthermia therapy^3^, etc. The most important physical quantities determining the transport are the transport coefficients representing the diffusivity, convectivity, reactivity/damping, and the sinks/sources in the system. Typically, these quantities cannot be measured directly but have to be inferred from measurements of the transport variable, such as the temperature for thermal transport or the concentration for particle transport. The evolution of the transport variable is described by the transport equation and is referred to as the state. Estimating the corresponding coefficients of the transport equation based on measurement is known as an inverse problem or referred to as grey box modelling.

In many cases, the geometry and the physics allow for the reduction of the transport model to a single spatial dimension. For example, in the context of magnetically confined plasmas there is a strong anistropy between transport processes along and perpendicular to the magnetic field lines. The toroidal geometery then allows for a 1D approach in the main plasma. In this context, a 1D approach has been proposed multiple times^4–7^ and is sometimes referred to as the matricial approach (MA). As pointed out by Escande and Sattin^7^, the main advantage of the MA is that after integration the exact value of two of the transport coefficients (using one frequency), namely diffusion and convection, can be determined without having to choose basis functions.

The MA is theoretically valid. However, experimental practicalities, such as the smoothing, extrapolation, and interpolation of the measurements, e.g. by splines, necessary to obtain acceptable spatial functions of the source, the state (and its derivatives) makes determination of the exact coefficients ambiguous. Smoothing operations on the (state) profile are equivalent to choosing lower order basis functions for the transport parameters in the estimation procedure. Hence, both are different forms of regularization and both can significantly impact the estimates of diffusion and convection. Moreover, the regularization of profile or transport parameters is a choice for optimization rather than an inherent modeling feature.

For estimation procedures, a strict distinction needs to be made between systems in which the source is at the system boundary and systems in which the source and sinks are within the domain. An example of the latter is a nuclear fusion reactor where there are various state dependent heating methods with state dependent source distributions. Here heating can stem from the nuclear fusion reaction as well as from neutral beam heating, ion cyclotron heating, electron cyclotron heating, lower hybrid heating, and Ohmic heating. To infer the transport in steady state, the power deposition for all these heating methods needs to be known in detail. To complicate matters, the heating method can be subject to, or even drive, plasma instabilities that lead to source broadening^8–14^. Therefore, the deposition width of sources within the domain may be uncertain or subject to dynamic changes. Hence, simultaneous estimation of the transport parameters and source profile is crucial to achieve reliable estimates and to ensure a broad application of these estimation methods. As the aforementioned methods do not deal with the simultaneous estimation of sinks/sources and transport coefficients, we developed a new approach allowing for this simultaneous estimation while we also provide transparency in the applied regularization. By using high order basis functions, no additional regularization is applied to the coefficients. Moreover, regularization on the level of the state by smoothing or interpolation is still an option.

Similarly to Ref.^7^, we formulate the problem as a linear regression problem which contains the source and damping. We use multiple harmonics because one of the strong advantages of estimation is over-determination, i.e., increasing the number of harmonics (equations) compared to the number of unknown coefficients reduces the uncertainty. To deal with this over-determination, we formulate the problem as a linear least squares problem for which we have a (unique) closed-form solution, thereby avoiding iterative optimization methods.

## Methodology

We consider the convection–diffusion–reaction equation often following from linearization

1 $$\begin{aligned} \partial _t z = -\nabla \cdot \left( {\textbf{V}}z -D \nabla z \right) + K z + P\phi , \end{aligned}$$

describing the spatio-temporal evolution of the perturbed transport quantity $z({\textbf{x}},t)$ where ${\textbf{x}}\in {\mathbb {X}}\subset {\mathbb {R}}^n$ denotes the spatial geometry. The transport parameters representing the diffusivity and reactivity are given by the functions $D({\textbf{x}})$ and $K({\textbf{x}})$, respectively. The diffusivity is a strictly positive function. For transport in multiple dimensions, the convectivity is a flow field given by the vector function ${\textbf{V}}({\textbf{x}})$, while for a single dimension it is given by the function $V({\textbf{x}})$. Furthermore, we consider an unknown source profile $P({\textbf{x}})$ that is fixed in space but modulated over time by the known signal $\phi (t)$. In our problem we measure $z({\textbf{x}},t)$, control $\phi (t)$, while the exact deposition $P({\textbf{x}})$ is unknown, and needs to be estimated in conjunction with the diffusivity *D*, flow velocity ${\textbf{V}}$, and reactivity *K*. Linear, but unknown boundary conditions constrain the edge of the spatial domain ${\mathbb {X}}$ and determine the solutions of the PDE (well-posedness). It is assumed that the initial condition of the problem is compatible with the model and its boundaries, but that the response due to the initial condition has already diminished or is compensated for through advanced signal processing, see Ref.^15^.

To estimate the transport coefficients $\lbrace D, {\textbf{V}}, K, P \rbrace$, we reformulate the methodology presented in Ref.^16^ to be applied to a more general *n*D setting. As the model, gradient and Laplacian operators are linear, we can rewrite (1) in the frequency domain as

2 $$\begin{aligned} i\omega Z = \left( \nabla ^2 Z \right) D + \nabla Z \cdot \nabla D - \nabla Z \cdot {\textbf{V}} - Z \left( \nabla \cdot {\textbf{V}}\right) + Z K + \Phi P, \end{aligned}$$

where $Z({\textbf{x}},\omega )$ and $\Phi (\omega )$ are the Fourier transform of $z({\textbf{x}},t)$ and $\phi (t)$, respectively. For now, consider that $\Phi$, *Z* and all of its derivatives are known such that the problem is linear in the unknown transport parameters, thus the resulting least squares problem is quadratic in the parameters^17^. However, as the transport parameters are functions of the spatial variable, the problem is infinite dimensional and hard to solve. For this reason, we apply a linear parametrization to each of the unknown parameters, say $\gamma \in \lbrace D, {\textbf{V}}, K, P \rbrace$. For this, introduce basis functions $B^\gamma ({\textbf{x}})$ and weighting vector $\theta ^\gamma$ of dimension $R^\gamma$ such that $\gamma = \sum _{r=1}^{R^\gamma } B_r^\gamma \theta ^\gamma _r$. This means that we only need to estimate $R = R^D + R^{\textbf{V}} + R^K + R^P$ weights that parameterize the functions. Furthermore, this parametrization allows us to rewrite the problem by grouping the known terms in vectors

3 $$\begin{aligned} G^D&= \begin{pmatrix} \left( \nabla ^2 Z \right) B^D_1 + \nabla Z \cdot \nabla B^D_1 \\ \vdots \\ \left( \nabla ^2 Z \right) B^D_{R^D} + \nabla Z \cdot \nabla B^D_{R^D} \end{pmatrix}^T, \end{aligned}$$

4 $$\begin{aligned} G^{\textbf{V}}&= \begin{pmatrix} - \nabla Z \cdot {\textbf{B}}^V_1 - Z \left( \nabla \cdot {\textbf{B}}^V_1\right) \\ \vdots \\ - \nabla Z \cdot {\textbf{B}}^V_{R^V} -Z \left( \nabla \cdot {\textbf{B}}^V_{R^V}\right) \end{pmatrix}^T,\end{aligned}$$

5 $$\begin{aligned} G^K&= \begin{pmatrix} Z B^K_1&\ldots&Z B^K_{R^K} \end{pmatrix}, \end{aligned}$$

6 $$\begin{aligned} G^P&= \begin{pmatrix} \Phi B^P_1&\ldots&\Phi B^P_{R^P} \end{pmatrix}, \end{aligned}$$

such that we can express (2) as an inner product

7 $$\begin{aligned} i\omega Z - \begin{pmatrix} G^D&G^{\textbf{V}}&G^K&G^P \end{pmatrix} \theta = 0, \end{aligned}$$

where $\theta$ stacks the vectors $\theta ^D, \ldots ,\theta ^P$ and should hold for all $({\textbf{x}},\omega ) \in {\mathbb {X}} \times \Omega$. As the user chooses the basis functions, the derivatives are known and (7) can be seen as a linear regression problem. Next, we approximate the equation error criterion^17^ by only considering the state *Z* at the measurement locations ${\textbf{x}} \in {\mathbb {X}}_{\mathbb {M}} \subset {\mathbb {X}}$ for the relevant frequencies $\omega \in \Omega _{{\mathbb {M}}}\subset \Omega$ that are well above the noise level (i.e. excited/perturbed). The error criterion is then given by

8 $$\begin{aligned} \begin{aligned} {\mathscr {V}}_{ee}&= \int _{\Omega } \int _{{\mathbb {X}}} \Big ( i\omega Z - \begin{pmatrix} G^D&G^{\textbf{V}}&G^K&G^P \end{pmatrix} \theta \Big )^2 \; \text{d}{\textbf{x}} \text{d}\varvec{\omega }\\&\approx \sum _{\Omega _{\mathbb {M}}} \sum _{{\mathbb {X}}_{\mathbb {M}}} \Big ( i\omega Z - \begin{pmatrix} G^D&G^{\textbf{V}}&G^K&G^P \end{pmatrix} \theta \Big )^2. \end{aligned} \end{aligned}$$

As the boundary conditions of the original problem are assumed to be unknown, the measurements on the edge of the measurement grid ${\mathbb {X}}_{\mathbb {M}}$ constitute Dirichlet boundary conditions, but other choices are feasible and can be added to the equation error^17^.

Similar to Refs.^7,16^, (8) can be seen as a linear regression model

9 $$\begin{aligned} {\bar{Y}} = {\bar{G}} \theta , \end{aligned}$$

where ${\bar{Y}}$ and ${\bar{G}}$ are a concatenation of the considered data points for $i\omega Z$ and $$\begin{pmatrix} G^D&G^{\textbf{V}}&G^K&G^P \end{pmatrix}$$, respectively. Then, the optimal solution in the least square sense is given by

10 $$\begin{aligned} \theta _{\text{opt}} = ({\bar{G}}^H {\bar{G}})^{-1}({\bar{G}}^H {\bar{Y}}), \end{aligned}$$

where ${\bar{G}}^H$ is the Hermitian transpose of ${\bar{G}}$. Hence, instead of iterative optimization procedures which may end up in local minima, we can simply evaluate the expression (10) to obtain the global minimum or use a linear equation solver. Moreover, due to linearity of the derived regression problem, different criteria than (8) such as weighted least squares also have a closed-form solution^18^ and are equally easy to implement and use. In addition, due to the linearity it is straightforward to extend this to more advanced estimators such as a maximum likelihood estimator^19^ which considers uncertainty in an optimal sense.

Until this point, we assumed that *Z*, $\nabla Z$ and $\nabla ^2 Z$ are known at the measured locations, but note that in practice only *Z* is measured at a limited number of spatial locations. Similar to Ref.^7^, the derivatives could be estimated by smoothing, interpolating and extrapolating the data set. By doing so, one forces a specific solution of the transport parameters as they are directly related to the interpolation method and its derivatives. For this reason, we approximate $\nabla ^2 Z$ and $\nabla Z$ using central finite difference^20,21^. For this semi-discretization it is known that increasing the number of (spatial) samples results in a convergence to the exact solution to the model^21^. As we have a closed-form solution for the global optimum, our solution will thus also converge to the exact solution for an increasing number of (spatial) samples, under the assumption that the selected basis functions of $\gamma$ have sufficient freedom. If the number of measurement locations is too limited such that it significantly affects the discretization accuracy, one can still resort to interpolation methods to increase the accuracy without requiring a complete continuous profile and therefore force a very specific solution.

Furthermore, the transport parameters are only considered at the data points that are included in the equation error. As a result, the estimated transport coefficients are exact at the considered data points if the provided derivatives are exact and the combination of basis functions gives sufficient freedom to describe the underlying function, while the values in-between data points can merely be used as interpolation of the estimated parameters. The maximum spatial variability of the transport coefficients is directly linked to the number of basis functions, i.e. free parameters, and the number of spatial data points that constraint these free parameters. Hence, if the number of basis functions for a coefficient is lower than the number of spatial data points minus two (for the boundary conditions), regularization is applied naturally. This is in contrast to the classical transport codes where the choice of basis functions directly limits the solution space. Moreover, due to the low computational cost of the closed form solution, many different parametrizations can be evaluated, such that a machine learning approach (as shown in Ref.^16^) can be applied to find a suitable parametrization.

## Simulation examples

### 1D simulation example

**Figure 1:**
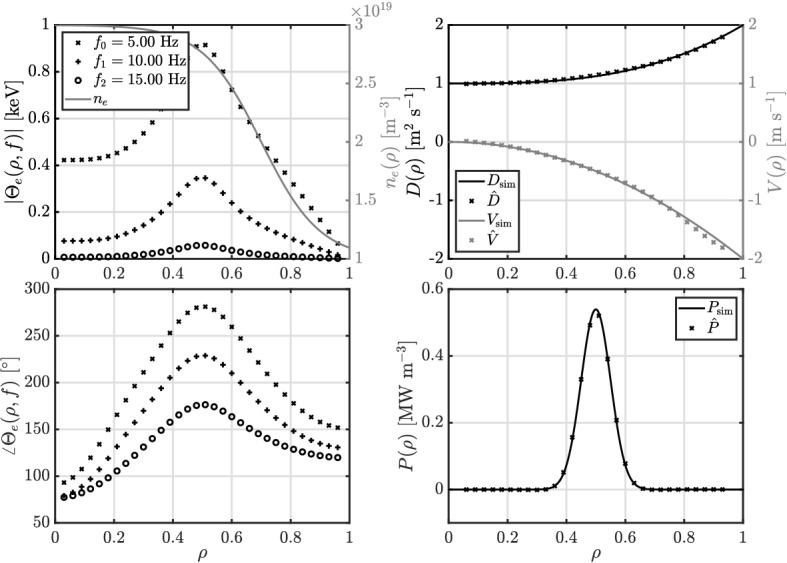
The density and simulated temperature profile (left) with the corresponding simulated and estimated transport parameters (right).

In order to demonstrate the presented methodology, we start with a 1D simulation for a fusion relevant scenario, where the electron temperature $\Theta _e$ in cylindrical geometry is described by

11 $$\begin{aligned} i\omega \frac{3}{2} n_e \Theta _e = \frac{1}{\rho } \partial _\rho \left( \rho D n_e \partial _{\rho } \Theta _e + \rho V n_e \Theta _e\right) + P \Phi, \end{aligned}$$

with spatially varying electron density $n_e(\rho )$. The temperature data is generated using a central finite-difference scheme with a grid ${\mathbb {X}}_{\mathbb {M}}$ of 1001 points and considering a block-wave modulation for $\phi$ with a 70% duty cycle at 5 Hz from which we only use the first three harmonics. The electron temperature is measured on an equidistant measurement grid consisting of 32 spatial points on which we assume the electron density and its gradient to be known. The simulated *D* and *V* are a third and second order polynomial, respectively, while the source profile *P* is a Gaussian. For the estimation procedure, $B^D$ and $B^V$ are sixth order polynomials and $B^P$ is a third order B-spline. In this way, $B^D$ and $B^V$ have more parameters than required to describe *D* and *V*. By matching the number of control points for $B^P$ with the spatial measurement grid minus two boundary conditions i.e. 30, no regularization is applied. The basis functions are multiplied with the required factors to account for the cylindrical geometry and spatially varying density, after which the unknown parameters are determined by evaluating (10). Figure 1 contains an overview of the used data and the estimation results. The estimated parameters are close to the simulated parameters despite the scarce finite difference grid to estimate the gradient. Only the estimate of *V* deviates somewhat at the end of the domain due to low amplitudes and change in density gradient.

**Figure 2:**
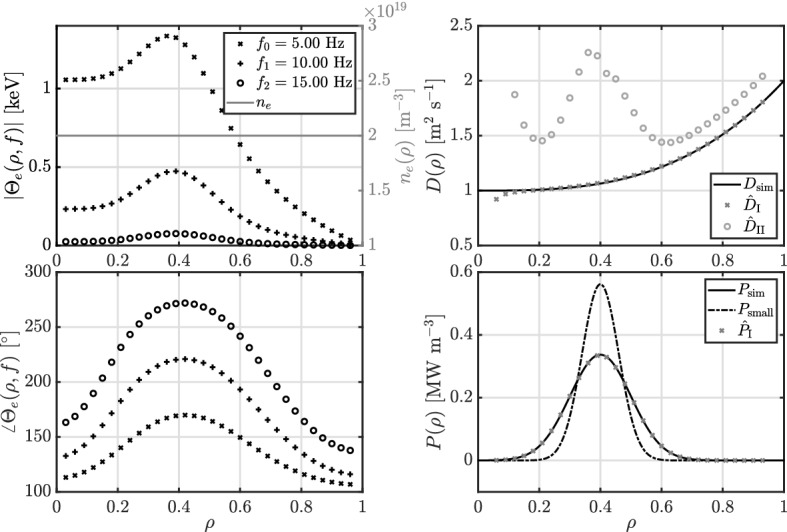
The density and simulated temperature profile (left) with the corresponding simulated and estimated transport parameters (right) for the two different scenario’s: (I) simultaneously estimate of the diffusivity ${\hat{D}}_\text{I}$ and source ${\hat{P}}_\text{I}$, and (II) estimate of the diffusivity ${\hat{D}}_\text{II}$ with the sources that is too narrow $P_\text{small}$.

As stated in Ref.^7^, the source is crucial to the parameter estimation problem. Therefore, we want to show the importance of simultaneously estimating the transport parameters and the source profile, as a small mismatch between the true and used source profile can result in a large difference in estimated transport parameters. For the sake of clarity, we consider (11) with a constant density (to avoid cross-error analysis), diffusivity described by a third order polynomial, no convection, and a Gaussian source profile. The diffusivity is now estimated for two different scenario’s: (I) simultaneously with the source profile; and (II) with a fixed source profile that is narrower than the simulated source profile. For the estimation, both diffusivity $B^D$ and source $B^P$ are third order B-spline with 30 control points such that there is no regularization. An overview of the used data and estimation results is given in Fig. 2.

In Fig. 2, scenario I), where the diffusivity and source are simultaneously estimated, there is only a small error in *D* at the boundary condition close to $\rho = 0$ due to low accuracy of the discretization grid. In Fig. 2, scenario II), with the fixed used source that is smaller than the true source, there is a large error on the diffusion in the region with the source and a bias in the region without source.

### 2D simulation example

Although our methodology is applicable in *n*D, for the sake of clarity and presentation, we demonstrate it on a 2D scenario. The 2D scenario mimics a rotating fluid that is heated by a modulated uniform and Gaussian profile and uniform diffusion over the entire domain. The transport coefficients are given by the following functions:

12 $$\begin{aligned} D(x,y)&= 1, \end{aligned}$$

13 $$\begin{aligned} {\textbf{V}}(x,y)&= \begin{pmatrix} u \\ v \end{pmatrix} = 500 \begin{pmatrix} 0.5-y\\ x-0.5 \end{pmatrix}, \end{aligned}$$

14 $$\begin{aligned} P(x,y)&= 1 + \frac{40}{\pi } e^{-\frac{\left( x-0.25\right) ^2}{0.1^2}} e^{-\frac{\left( y-0.5\right) ^2}{0.1^2}}. \end{aligned}$$

**Figure 3:**
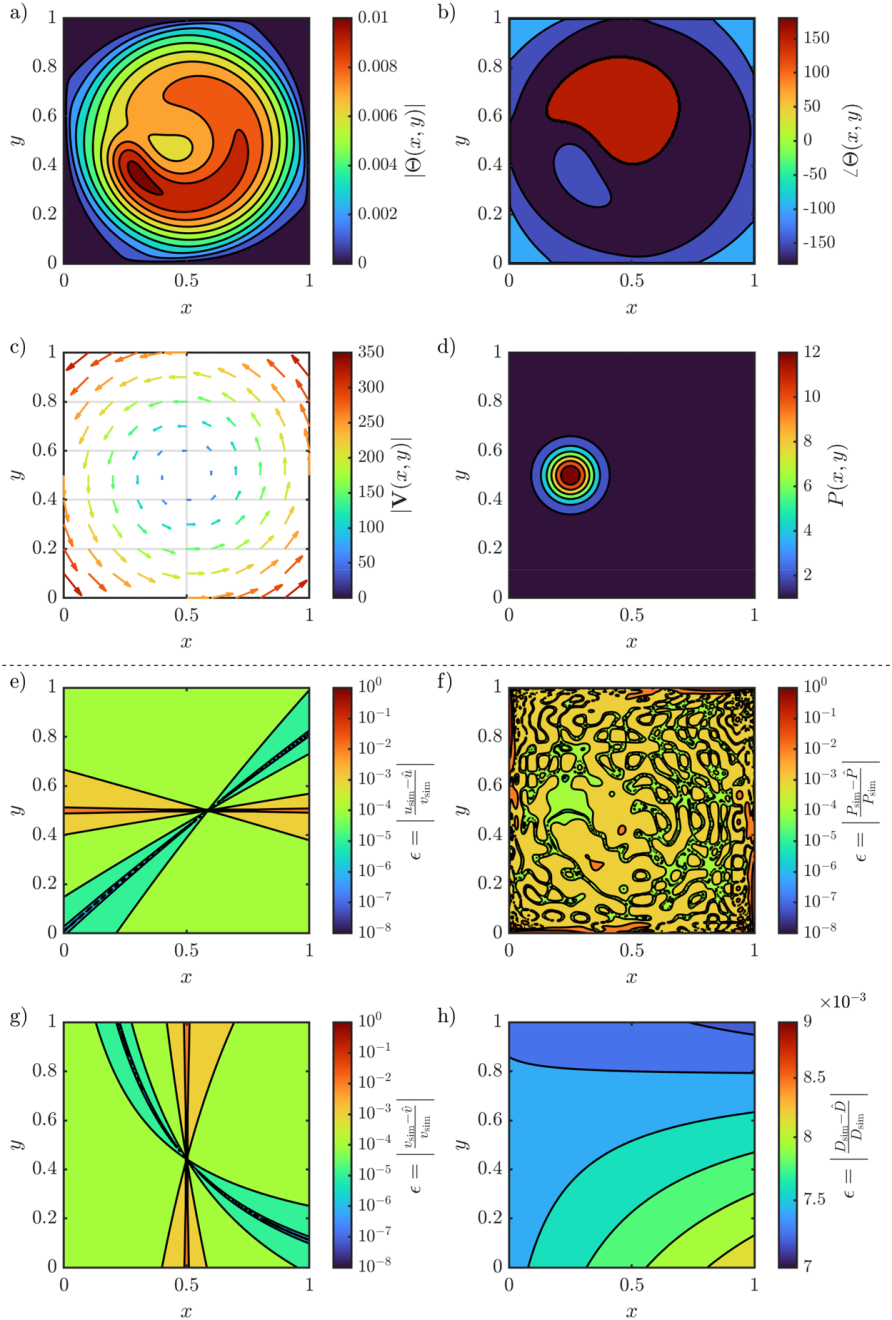
The 2D scenario with the absolute temperature profile of the modulation frequency at 25 Hz (**a**) and the phase (**b**), followed by the flow field (**c**) and source deposition profile (**d**). The relative estimation errors are shown for the x-component (e) and y-component (**g**) of the flow field, the source (**f**) and diffusivity (**h**).

The source is modulated by a 25 Hz block wave with a 50% duty cycle, where we only consider the first three harmonics (25 Hz, 75 Hz, 125 Hz). The temperature profile is generated by solving (2) using the finite volume-complete flux scheme^22^ with a grid of $1001 \times 1001$ points, and the measurements are taken on a $251 \times 251$ grid for the estimation procedure. The corresponding temperature profile of the first harmonic, the used convection and source are visualized in the four top subfigures in Fig. 3.

The unknown 2D transport coefficients are parameterized by taking the product of two 1D basis functions for each $\gamma \in \lbrace D, u, v, K, P\rbrace$,

15 $$\begin{aligned} B_r^\gamma (x,y) = B_{r_x}(x) B_{r_y}(y), \end{aligned}$$

with $r^\gamma = r^\gamma _x + (r^\gamma _y-1) R^\gamma _y$, where $R^\gamma _y$ is the total number of basis functions in the *y* direction. Here, $B^D$, $B^u$, $B^v$ are $2 \times 2$ Chebyshev polynomials of the first kind and $B^P$ a $40 \times 40$ Chebyshev polynomial. Hence, in total, we need to estimate 1612 unknown parameters, where evaluating (10) takes approximately 100 ms on a regular desktop. The estimation error on each function is also shown in Fig. 3, which clearly shows that the parameters are well estimated and only small errors are made with respect to the function values. The error on *D*, *u* and *v* are low order polynomials due to the low order of the approximation, while the error on *P* is the result of Chebyshev polynomials that cannot perfectly describe the Gaussian deposition profile and compensation for the errors made by the finite-difference method for the gradient approximation. Note that we display the (log) relative error. As *u* and *v* cross zero at 0.5, the relative error tends to infinity and is therefore large close to 0.5 while being small in the other regions.

## Conclusion and discussion

In conclusion, our proposed methodology provides new opportunities to study *n*D transport by estimating the sinks/sources and transport coefficients while providing transparency about regularization and interpolation with extremely low computational effort due to the closed-form solution that guarantees the global optimum for the selected optimization criteria. The low computational cost of the closed-form solution opens up a number of new significant opportunities: (i) machine learning like approaches to find the best parameterization of the transport coefficients as demonstrated in Ref.^16^; (ii) fast estimation of multidimensional transport coefficients, for which we present the first results in this letter. Moreover, as the source no longer needs to be localized, since the transport parameters and sources/sinks can be estimated simultaneously, adding a source to regions with a low signal-to-noise can significantly improve the quality of the coefficient estimation.

Furthermore, it is possible to extend the current methodology to include more coefficients, e.g., derivatives to other variables following from linearization or cross-terms from coupled transport. However, there is no guarantee that these additional coefficients can be estimated by simply considering more harmonics as the solution to the inverse problem with additional coefficient functions is not necessary unique. This is in contrast to the considered diffusion–convection–reaction-source equation which is known to have a unique inverse solution. Hence, uniqueness of solution should be studied before considering more coefficients.

Finally, the proposed methodology does not consider noise. In case of noisy measurements, the estimation problem becomes an error-in-variables problem as the measurements are used in the regressor matrix. Therefore, estimates with the proposed least squares estimator will be biased where the bias depends on the signal-to-noise ratio. Moreover, the gradients in our proposed methodology are computed via a finite difference scheme. Therefore, the uncertainty of the estimated gradients is a combination of the uncertainty of the surrounding sensors and scales with the distance between sensors. Hence, denser measurement grids will increase the uncertainty on the estimated gradients.

The best way to deal with noise is to use an estimator that takes the noise into account, e.g., Bayesian or maximum likelihood. For transport 1D this is still computable (e.g., see Ref.^19^), whereas for *n*D transport this quickly becomes computational expensive due to the curse of dimensionality that applies to both the data set and the number of free parameters. Therefore, a computational better, but less optimal way to deal with noise is to use a weighted (total) least squares criterion with a closed-form solution where the weights are based on the uncertainty. Furthermore, the effect of noise in the finite-difference gradient approximation can be mitigated by smoothing the measurements and using the smoothing function to estimate the gradients, e.g via spline interpolation which is common practice in some fields^7,10^. In the estimation of *n*D transport coefficients there is clear tradeoff between accuracy and computational cost. The effect of noise on the estimates with different criterion and new methods to efficiently deal with noise in *n*D transport are part of our future research.

## Data Availability

The data and code that supports the findings of this study and are used to generate the figures are available upon reasonable request from the corresponding author.
